# The role of extended-release niacin on immune activation and neurocognition in HIV-infected patients treated with antiretroviral therapy – CTN PT006: study protocol for a randomized controlled trial

**DOI:** 10.1186/1745-6215-15-390

**Published:** 2014-10-07

**Authors:** Bertrand Lebouché, Mohammad-Ali Jenabian, Joel Singer, Gina M Graziani, Kim Engler, Benoit Trottier, Réjean Thomas, Marie-Josée Brouillette, Jean-Pierre Routy

**Affiliations:** Chronic Viral Illness Service, Montreal Chest Institute, McGill University Health Centre, 3650 Saint Urbain St., Montreal, QC H2X 2P4 Canada; Canadian Institutes of Health Research (CIHR) Canadian HIV Trials Network (the CTN), 588-1081 Burrard St., Vancouver, BC V6B 3E6 Canada; Department of Family Medicine, McGill University, 5858, chemin de la Côte-des-Neiges, Montreal, QC H3S 1Z1 Canada; Ottawa Hospital Research Institute, 501 Smyth Rd., Ottawa, ON K1H 8L6 Canada; Clinique médicale l’Actuel, 1001 boul. de Maisonneuve E, Montreal, QC H2L 4P9 Canada

**Keywords:** Antiretroviral therapy (ART), Disease progression, Extended-release (ER) niacin, Human immunodeficiency virus (HIV-1), T-cell immune activation, Tryptophan (Trp), Vitamin B3

## Abstract

**Background:**

Approximately 30% of HIV-1-infected patients receiving antiretroviral therapy who achieve virologic control have unsatisfactory immune reconstitution, with CD4+ T-cell counts persistently below 350 cells/μL. These patients are at elevated risk for clinical progression to AIDS and non-AIDS events. CD4+ T-cell depletion following infection and persistent immune activation can partially explain this low CD4+ T-cell recovery. Recent data suggest a link between the tryptophan oxidation pathway, immune activation and HIV disease progression based on overstimulation of the tryptophan oxidation pathway by HIV antigens and by interferon-gamma. This overstimulation reduces levels of circulating tryptophan, resulting in inflammation which has been implicated in the development of neurocognitive dysfunction. Niacin (vitamin B3) is able to control the excess tryptophan oxidation, correcting tryptophan depletion, and therefore represents an interesting strategy to improve CD4 recovery.

We aim to design a crossover proof-of-concept study to assess supplementation with an extended-release form of niacin (Niaspan FCT™) in combination with antiretroviral therapy, compared to antiretroviral therapy alone, on T-cell immune activation as defined by changes in the percentage of CD8+ CD38+ HLA-DR+ T-cells.

**Methods/Design:**

This randomized, open-label, interventional crossover study with an immediate versus deferred use of Niaspan FCT for 24 weeks will assess its ability to reduce immune activation and thus increase CD4 recovery in 20 HIV-infected individuals with suboptimal immune responses despite sustained virologic suppression. A substudy evaluating neurocognitive function will also be conducted.

**Discussion:**

This randomized trial will provide an opportunity to evaluate the potential benefit of oral extended-release niacin, a drug that can indirectly increase tryptophan, to reduce immune activation and in turn increase CD4+ T-cell recovery. The study will also allow for the evaluation of the impact of Niaspan FCT on neurocognitive function in HIV-infected individuals with suboptimal immune responses despite sustained virologic suppression.

**Trial registration:**

This study was registered with ClinicalTrials.gov on 17 December 2013 (registration number: NCT02018965).

**Electronic supplementary material:**

The online version of this article (doi:10.1186/1745-6215-15-390) contains supplementary material, which is available to authorized users.

## Background

Approximately 30% of antiretroviral therapy (ART)-treated patients who achieve virologic control have an unsatisfactory immune reconstitution, with CD4+ T-cell counts persistently below 350 cells/μL [1, 2]. These patients remain at an elevated risk for clinical progression to both AIDS and non-AIDS events [1, 3–5]. Depending on the definition, it is estimated that between 5 and 20% of patients starting ART can be considered as immunological non-responders. These individuals have suboptimal CD4+ T-cell recovery despite having complete suppression of their plasma viral load [6, 7]. Accumulating data suggest that CD4+ T-cell depletion following infection, as well as persistent immune activation, can partially explain this low and/or slow CD4+ T-cell recovery [1, 8, 9]. Indeed, in patients receiving effective ART, the persistence of enhanced T-cell activation (defined by co-expression of CD38 and HLA-DR on CD8+ T-cells) and elevated plasma levels of markers of inflammation, such as interleukin (IL)-6, C-reactive protein (CRP) and D-dimers, represent factors associated with low CD4+ T-cell recovery [10–12]. In addition, persistent immune activation leads to a chronic inflammatory state that is associated with an increased risk of the non-opportunistic complications that are observed in ART-treated individuals, such as cardiovascular, renal and hepatic events [13, 14].

Recent data suggest a link between the tryptophan (Trp) oxidation (TrpOx) pathway (also known as the Trp catabolism pathway), immune activation and HIV disease progression [15, 16]. Indoleamine 2,3-dioxygenase (IDO) expressed by dendritic cells and macrophages catabolizes the essential amino acid Trp into an immunosuppressive metabolite kynurenine (Kyn) that limits immune responses [17]. It has been reported that IDO mediates the loss of IL-17-secreting (Th17) cells that enhance host defenses against microbial pathogens, mainly at mucosal barrier sites [15]. As a result of the disturbance in the balance between Th17 and immunosuppressive T-regulatory (Treg) cells, there is a breakdown of the mucosal barrier. This breakdown causes increased levels of circulating microbial products, such as lipopolysaccharides (LPS), which sustain chronic immune activation [18, 19].

Trp is an essential amino acid required for the biosynthesis of proteins, serotonin and niacin, which is a vitamin B3 compound [20]. Both HIV antigens and interferon-gamma (IFN-γ) can overstimulate the TrpOx pathway by activating IDO, resulting in Trp catabolism into Kyn [15]. As IDO is the rate-limiting enzyme in the metabolism of Trp along the Kyn pathway, circulating levels of Trp are reduced in HIV-infected patients despite adequate dietary intake [16]. Following Trp catabolism into Kyn under the influence of IDO, there is an additional catabolic pathway in which the metabolites contribute to an imbalance between neuroprotective and neurotoxic metabolites in the Kyn pathway [21, 22]. This occurs when Kyn is further catabolized by the enzyme kynurenine-3-mono-oxygenase into 3-hydroxy-Kyn (3-OHK) and the N-methyl-D - aspartate (NMDA) receptor agonist quinolinic acid. 3-OHK causes neuronal apoptosis while quinolinic acid causes excitotoxic neurodegenerative changes. Moreover, quinolinic acid has pro-inflammatory properties [23], further stimulating IDO and thus aggravating the inflammatory response and Trp degradation. However, Kyn can also be catabolized into kynurenic acid, which is an NMDA receptor antagonist and is protective against the excitotoxic action of quinolinic acid [24]. Evidence linking the Kyn pathway and cognitive dysfunction has been recently reviewed by Davies *et al*. [21]; both direct and indirect evidence supports its role in Alzheimer’s disease as well as in HIV-associated neurocognitive disorders (HANDs). Thus, it is plausible that modifying Trp metabolism along the Kyn pathway may improve HANDs.

*In vitro* data has shown that Trp depletion can significantly impede T-cell reconstitution by reducing both CD4+ T-cell proliferation and survival [25, 26]. ART is able to partially decrease immune activation through viral load suppression while normalizing Trp plasma levels and decreasing Trp catabolism into Kyn [27–29]. Ongoing IDO activation also appears to contribute significantly to the development of several symptoms known to deteriorate a patient’s quality of life (QoL). These include the neuropsychiatric symptoms of fatigue, anemia and weight loss experienced by HIV and cancer patients [30, 31].

Collectively, these data strongly suggest that declining Trp levels are associated with immunosuppression, mood disturbances and cognitive impairment, thereby leading to the hypothesis that oral Trp supplementation should contribute to improved immune responses and cognitive function. However, in clinical studies, direct Trp supplementation in the presence of ongoing immune activation was associated with increased risks of neurotoxic problems, oxidative stress and possibly the development of eosinophilia-myalgia syndrome, a rare and deadly flu-like condition [32, 33].

An indirect way to increase Trp levels while minimizing the side effects of direct Trp supplementation is to provide exogenous niacin, a key player in the TrpOx pathway. Niacin, also known as vitamin B3, nicotinic acid and vitamin PP, is able to control excess Trp oxidation by a process of reverse inhibition [34]. It has been shown that exogenous niacin supplementation in the form of nicotinamide, the major circulating form of niacin, is able to correct Trp depletion and improve CD4+ T-cell recovery [35, 36]. Improvements in cognitive test scores and overall function have been reported in European trials of pharmacological preparations that include niacin [37].

In one small study, four ART-naïve HIV-infected individuals were treated with a supplement of 3,000 mg/day of immediate-release (IR) niacin for two months. The treatment was not associated with the usual side effects such as flushing (described as warmth), redness, itching and/or tingling, and resulted in a 40% increase in plasma Trp levels and clinical benefits [36]. However, further studies on the effectiveness of niacin to increase Trp levels are needed to confirm these results.

Niaspan FCT™ is an extended-release (ER), oral form of niacin that is dosed once each night when cholesterol synthesis and fatty acid mobilization are at their peak. Niaspan FCT has intermediate-release characteristics and is thus designed to minimize both liver enzyme elevation and flushing [38].

Niacin is the most effective agent at raising high-density lipoprotein cholesterol levels, either as a monotherapy or in combination with other agents, despite the fact that it has not consistently shown improved patient outcomes [39]. However, it has been recently shown that in patients with atherosclerotic cardiovascular disease and low-density lipoprotein cholesterol levels, there was no incremental clinical benefit from the addition of niacin to statin therapy, despite significant improvements in high-density lipoprotein cholesterol and triglyceride levels. We will, therefore, also assess the ability of Niaspan FCT to improve cholesterol and triglyceride levels [40].

Since the beginning of the AIDS epidemic, HANDs have been commonly observed in individuals in the advanced stage of the disease [21]. More recently, age, cerebrovascular diseases and the Kyn pathway and immune activation have also been suggested to be involved in HANDs [21, 22, 41]. Low nadir CD4 cell count and low CD4 recovery have also been reported in many studies as risk factors for prevalent neurocognitive impairments [42, 43].

Schroecksnadel *et al*. reported that enhanced Trp degradation was associated with impaired QoL in patients with cancer [30]. Collectively, these data strongly suggest that enhanced Trp degradation by persistent immune activation may provide an explanation for HANDs in HIV-infected patients despite treatment with ART [21, 22].

Given that ER niacin can indirectly increase Trp levels, we seek to further assess the effects of ER niacin on neurocognitive functions and QoL among HIV-infected individuals who choose to participate in a substudy of this clinical trial.

This pilot crossover study will be conducted to examine the ability of ER niacin to reduce immune activation, which may in turn increase CD4 recovery in HIV-infected individuals with suboptimal immune responses despite sustained virologic suppression. The main hypothesis to be tested is that ER niacin co-administration with ART will be associated with decreased T-cell activation as determined by changes in the percentage of activated CD8+ CD38+ HLA-DR+ T-cells. If so, during administration of ER niacin, participants should experience a time-dependent decrease in T-cell activation, and such a decrease may reverse after ER niacin is discontinued. We will also examine the effects of ER niacin on cognition. To this end, we will assess the neurocognitive function and QoL in participants using standardized neuropsychological (NP) tests and questionnaires.

## Methods/Design

### Study design

**Figure 1:**
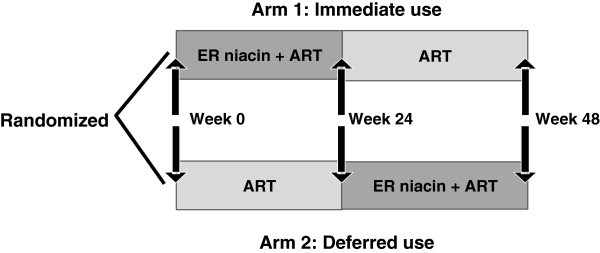
**Study design.** Arm 1: ER niacin administration begins at week 0 and ends at week 24 (defined as ‘immediate use’ arm). Arm 2: ER niacin administration begins after the week 24 visit and ends at week 48 (defined as ‘deferred use’ arm). ART, Antiretroviral therapy; ER, extended-release.

This is a randomized, open-label, interventional crossover study (the Canadian HIV Trials Network (CTN) number PT006) with an immediate versus deferred use of ER niacin for 24 weeks to assess the ability of oral ER niacin to reduce immune activation (as determined by the percentage of activated CD8+ CD38+ HLA-DR+ T-cells), which may in turn increase CD4+ T-cell recovery in HIV-infected individuals who achieve suboptimal immune responses despite sustained virologic suppression. Participants will continue to take their ART treatment as prescribed throughout the study. The study design is shown in Figure 1.

### Setting

Participants will be recruited at the Chronic Viral Illness Service, Montreal Chest Institute of the McGill University Health Centre (MUHC), one of the largest hospital-based centers in Montreal, Quebec, Canada, and at the Clinique médicale l’Actuel, the largest private medical centre in Montreal.

### Recruitment and enrollment

Prior to physical contact with any patient by the study staff, the HIV specialist at each HIV clinic will conduct chart reviews of prospective HIV-positive participants ahead of their scheduled clinic visits to determine which patients successfully treated with ART did not complete immune restoration. Based on this information, the research nurse and/or the referring HIV specialist will approach potential trial participants upon arrival at the clinics. At this time, the trial staff will inform the patient about the trial and invite him or her for eligibility screening and possible enrolment into the trial. Prior to enrolment, trial participation eligibility will be documented, and written informed consent will be obtained from eligible patients by the treating physician (Please see Additional file 1). Individuals who wish to discuss their participation in the study with their treating physician and/or with their family are given an opportunity to do so and may enrol at their next scheduled clinic visit. After enrolment, all participants will be followed during this trial by the principal investigator at the Chronic Viral Illness Service. In cases where the patient lacks capacity, a legal representative will provide consent.

#### Inclusion criteria

Eligible participants must meet all of the following criteria within four weeks prior to the week 0 (baseline) visit to be considered eligible for entry into the study: 1) documented HIV infection by western blot, enzyme immunoassays or viral load assay; 2) aged 21 years or older; 3) viral load <50 copies/mL for the last three months; 4) CD4+ T-cell count ≤350 cells/μL; and 5) on stable ART (ART unchanged for treatment failure (rebound in viral load)) for more than 12 months.

#### Exclusion criteria

Subjects who meet any of the following criteria will be excluded: 1) prior history of hypersensitivity reaction to niacin or any other component of the study drug; 2) prior history of flushing; 3) liver disease (including co-infection with hepatitis B or C virus) or unexplained persistent elevations of serum transaminases; 4) alanine aminotransferase (ALT) or aspartate aminotransferase (AST) or alkaline phosphatase >2.5 times the upper limit of normal (ULN); 5) active duodenal or gastric peptic ulcer; 6) active bleeding disorders; 7) history of gout; 8) active AIDS events in the last three months as determined by the treating physician; 9) unstable angina or acute phase myocardial infarction; 10) diabetic or potentially diabetic with hypercholesterolemia; 11) renal dysfunction; 12) co-enrollment in another study involving neurocognitive evaluation; or 13) pregnant or nursing or planning to become pregnant. Infection with syphilis, for which testing is done at screening, is not an exclusion criterion if the patient is treated; participants will be tested again at weeks 24 and 48.

### Study intervention

The study medication is ER niacin (Niaspan FCT; Sunovion Pharmaceuticals Canada Inc., Mississauga, Ontario, Canada) 500 and 1,000 mg ER film coated tablets (FCT) which will be taken at bedtime. The dose for this study is that recommended in the Niaspan FCT product monograph for the treatment of dyslipidemia [44]. ER niacin will be administered as outlined in Tables 1 and 2.

**Table 1:** **Arm 1 study drug administration schedule**

	Weeks	Daily dose	Niaspan FCT dosage
Initial titration schedule	0 to 4	500 mg	One 500 mg tablet at bedtime
	5 to 8	1,000 mg	One 1,000 mg tablet or two 500 mg tablets at bedtime
	9 to 12	1,500 mg	One 1,000 mg tablet and one 500 mg tablet at bedtime
	13 to 24	2,000 mg	Two 1,000 mg tablets at bedtime

**Table 2:** **Arm 2 study drug administration schedule**

	Weeks	Daily dose	Niaspan FCT dosage
Initial titration schedule	25* to 28	500 mg	One 500 mg tablet at bedtime
	29 to 32	1,000 mg	One 1,000 mg tablet or two 500 mg tablets at bedtime
	33 to 36	1,500 mg	One 1,000 mg tablet and one 500 mg tablet at bedtime
	37 to 48	2,000 mg	Two 1,000 mg tablets at bedtime

*Arm 2 extended-release niacin administration starts after the week 24 visit.

### Randomization

Once a participant is deemed eligible and has provided consent, they will be randomized to one of two treatment arms in a 1:1 ratio to receive the immediate or the deferred intervention with ER niacin. Prior to the initiation of the trial, a CTN staff member not directly associated with the implementation of the trial will use an SAS module (SAS Institute, Cary, North Carolina, United States) to generate sequential participant study identification numbers which will be loaded onto a webpage randomization system. The treatment groups will be: arm 1: ER niacin supplementation for 24 weeks followed by 24 weeks of ART alone, and arm 2: 24 weeks of ART alone followed by ER niacin supplementation for 24 weeks. When a participant is deemed eligible, the study coordinator signs onto the password-protected randomization webpage which issues the allocation. A transaction file records the study identification number, allocation and date and time of randomization.

Scheduled visits for arm 1 of this study will be screening (up to four weeks prior to treatment), week 0 (baseline), week 4, week 8, week 12, week 16, week 20, week 24, and week 48/final visit (or other if terminating early). Table 3 shows the arm 1 schedule of study events.

**Table 3:** **Visit schedule, arm 1**

	Screening	Week 0	Week 4	Week 8	Week 12	Week 16	Week 20	Week 24	Week 48 (final visit)
Visit number	V1	V2	V3	V4	V5	V6	V7	V8	V9
Consent form	X								
Medical history	X								
Dietary survey		X							
Physical exam and vital signs	X	X	X	X	X	X	X	X	X
Adverse events		X	X	X	X	X	X	X	X
Hematology	X	X	X	X	X	X	X	X	X
Chemistry	X	X	X	X	X	X	X	X	X
Pregnancy test (women only)	X								
Hepatitis B and C tests	X								
ART review	X								
Syphilis test^1^	X							X	X
T-cell activation and inflammatory markers		X						X	X
HIV viral load^2^	X				X			X	X
Levels of tryptophan, niacin and its derivatives		X		X		X		X	X
ER niacin admin^3^		X	X	X	X	X	X	X	
Bays-Ballantyne questionnaire			X	X	X	X	X	X	
WHOQOL-HIV BREF, CES-D, POMS, HVLT-R™, and CANTAB™ tests^4^		X						X	X
SMAQ^5^		X			X			X	X

^1^If Syphilis test is positive at screening, participants will be retested at weeks 24 and 48.

^2^HIV viral load testing will be performed as indicated by treating physician.

^3^Participants will end ER niacin administration at the end of week 24.

^4^Neurocognitive assessments are optional; WHOQOL-HIV BREF, World Health Organization quality of life instrument in patients with HIV infection-brief; CES-D, Center for Epidemiologic Studies Depression Scale; POMS, Profile of Mood State; HVLT-R™, Hopkins Verbal Learning Test Revised; CANTAB, Cambridge Neuropsychological Test Automated Battery.

^5^SMAQ, Simple Medication Adherence Questionnaire.

Scheduled visits for arm 2 of this study will be screening (up to four weeks prior to study enrolment), week 0 (baseline), week 24, week 28, week 32, week 36, week 40, week 44 and week 48/final visit (or other if terminating early). Table 4 shows the arm 2 schedule of study events.

**Table 4:** **Visit schedule, arm 2**

	Screening	Week 0	Week 24	Week 28	Week 32	Week 36	Week 40	Week 44	Week 48 (final visit)
Visit number	V1	V2	V3	V4	V5	V6	V7	V8	V9
Consent form	X								
Medical history	X								
Dietary survey		X							
Physical exam and vital signs	X	X	X	X	X	X	X	X	X
Adverse events		X	X	X	X	X	X	X	X
Hematology	X	X	X	X	X	X	X	X	X
Chemistry	X	X	X	X	X	X	X	X	X
Pregnancy test (women only)	X		X						
Hepatitis B and C tests	X								
ART review	X								
Syphilis test^1^	X		X						X
T-cell activation and inflammatory markers		X	X						X
HIV viral load^2^	X		X			X			X
Levels of tryptophan, niacin and its derivatives		X	X		X		X		X
ER niacin admin^3^				X	X	X	X	X	X
Bays-Ballantyne questionnaire				X	X	X	X	X	X
WHOQOL-HIV BREF, CES-D, POMS, HVLT-R™, and CANTAB™ tests^4^		X	X						X
SMAQ^5^		X	X			X			X

^1^If syphilis test is positive at screening, participants will be retested at weeks 24 and 48.

^2^HIV viral load testing will be performed as indicated by treating physician.

^3^Participants will start ER niacin administration after their week 24 visit.

^4^Neurocognitive assessments are optional; WHOQOL-HIV BREF, World Health Organization quality of life instrument in patients with HIV infection-brief; CES-D, Center for Epidemiologic Studies Depression Scale; POMS, Profile of Mood State; HVLT-R™, Hopkins Verbal Learning Test Revised; CANTAB, Cambridge Neuropsychological Test Automated Battery.

^5^SMAQ, Simple Medication Adherence Questionnaire.

### Measurements

At baseline, the following clinical information will be collected for all participants: age, education, ethnicity, handedness, communication skills (able to communicate in English and/or French adequately, first language), list of current psychoactive medication, dosage and date of treatment initiation, psychiatric disorders, duration of HIV infection, current ART regimen (duration in months), review of ART history, CD4+ T-cell count within the past three months, nadir CD4+ T-cell count, HIV plasma viral load within the past three months and any preexisting medical conditions, signs, or symptoms.

### Clinical parameters

Assessments performed at scheduled study visits will monitor safety (physical exam with vital signs, occurrence of adverse events (AEs and concomitant medications) and will determine the dermatological effects of flushing. At the follow-up visits, some or all of the following assessments will be done: plasma HIV RNA levels, hematology, fasted biochemistry, CD4+ T-cell and CD8+ T-cell counts, plasma levels of Trp and Kyn, T-cell activation and inflammatory markers and testing for syphilis if the participant tested positive at baseline (week 0).

### Dietary survey

A dietary survey to determine the pre-study dietary niacin levels for each participant will be completed at week 0 (baseline). This survey will be performed by an MUHC dietician with expertise in HIV patient management. Participants will be instructed to keep their diet constant during the study.

### Laboratory investigations

#### Medication adherence

Adherence for both ER niacin and ART will be measured using the Simple Medication Adherence Questionnaire (SMAQ) [45]. This questionnaire consists of seven binary questions regarding adherence during the three previous months. The SMAQ score ranges from 0 to 7, with 0 corresponding to 100% adherence.

### Optional neurocognitive assessments: quality of life, depression and neurocognitive scores

QoL will be assessed with the World Health Organization quality of life instrument in patients with HIV infection brief (WHOQOL-HIV BREF) [46], an instrument that provides a reliable and valid assessment of wellbeing in people living with HIV and AIDS. This 31-item assessment has very good discriminant validity with reference to ‘known’ disease groups, and generally very good internal consistency reliability.

Mood will be assessed by the Center for Epidemiologic Studies Depression scale (CES-D), and the Profile of Mood State (POMS) questionnaire. The CES-D is a 20-item short self-report scale designed to measure depressive symptomatology in the general population [47]. It has very high internal consistency and adequate test-retest repeatability. The POMS will measure depression as well as other mood states, irritability in particular. It is composed of six scales: agreeable-hostile, composed-anxious, elated-depressed, confident-unsure, energetic-tired and clearheaded-confused. It is highly sensitive to non-clinical changes in mood states [48].

Changes in cognition will be assessed with the Hopkins Verbal Learning Test Revised (HVLT-R™) and the Cambridge Neuropsychological Test Automated Battery (CANTAB™). The HVLT-R is a pencil and paper test that explores verbal episodic memory, learning, recall and verbal recognition processes [49]. The CANTAB is a computer-based cognitive assessment system; the computerized tests do not present any limitations due to language [50]. Eight subtests have been selected as representing the cognitive domains which are more at risk of decline in HIV infection: motor screening, rapid visual information processing, spatial working memory, paired associate learning, big/little circle, intra-extra dimensional set shift, stop signal task and Stockings of Cambridge task. The neuropsychological tests will be performed at weeks 0, 24 and 48 only on participants who consent to the optional neurocognitive evaluation. The entire testing battery will be administered by a trained research assistant under the supervision of a neuropsychologist. The total assessment time (including tests and questionnaires) will be approximately two hours and 30 minutes per session.

### Research hypothesis

The main hypothesis to be tested in this study is that ER niacin co-administration with ART will be associated with decreased immunosuppressive Trp catabolism and T-cell activation as determined by the proportion of activated CD8+ CD38+ HLA-DR+ T-cells.

### Study outcome measures

The primary objective of this study will be to assess the impact of ER niacin supplementation in combination with ART compared to ART alone on T-cell immune activation as defined by changes in the percentage of CD8+ CD38+ HLA-DR+ T-cells.

The secondary objectives of this study will be to: 1) assess the change in total CD4+ T-cell count after ER niacin administration; 2) explore the effect of ER niacin on Th17 and Treg cell frequencies in blood; 3) explore the effect of ER niacin on cytokines and inflammatory markers such as interferon-alpha (IFN-α), IL-1, IL-6 and IL-17, interferon gamma-induced protein 10 (IP-10), D-dimers, highly-sensitive C-reactive protein (hsCRP) and LPS; 4) assess the influence of ER niacin on Trp plasma levels; 5) assess changes in cholesterol and triglyceride levels; 6) evaluate the impact of ER niacin on QoL, fatigue, depression and neurocognitive scores; and 7) explore ER niacin tolerance.

### Efficacy variables

#### T-cell activation assessments

The primary endpoint of this study will be changes in the percentage of CD8+ CD38+ HLA-DR+ T-cells which will be used as a marker for the change in the level of T-cell activation. Enumeration of the CD38 activation marker on total CD4+ and CD8+ T-cells, as well as on naïve, memory and effector cell subsets, will be performed by eight-color flow cytometry. This will allow for the precise identification of which cell subpopulation(s) is or are more likely to downregulate CD38 expression following ER niacin administration.

The absolute numbers of activated T-cell subsets will be computed using: 1) the white blood cell (WBC) count; 2) the percentage of lymphocytes; 3) the percentage of CD4+ and CD8+ T-cells; and 4) the percentage of each CD4+ or CD8+ T-cell subset (naïve, central memory, pre-terminal effector memory or terminal effector memory).

#### T-cell activation in blood

Levels of T-cell activation will be assessed in blood samples on weeks 0, 24 and 48. This analysis will be performed using specific monoclonal antibodies (mAbs) as previously reported [51, 52]. Briefly, peripheral blood mononuclear cells (PBMCs) will be obtained by Ficoll-Hypaque density gradient centrifugation. The PBMCs will be either stained with the appropriate mAbs or stored in liquid nitrogen until used. T-cell subsets will be identified using a mAb cocktail specific for CD3, CD4, CD8, CD45RA, CCR7, CD27, CD38 and HLA-DR. One million PBMCs will be incubated in a 96-well polystyrene plate (Fisher Scientific, Ottawa, Ontario, Canada) in the dark at 4°C for 30 minutes. The cells will be washed twice with phosphate buffer saline (Fisher Scientific, Ottawa, Ontario, Canada) containing 1% fetal calf serum (Sigma Aldrich, Oakville, Ontario, Canada), resuspended in 250 μL 2% paraformaldehyde (Sigma Aldrich, Oakville, Ontario, Canada) and stored at 4°C for up to 12 hours before flow cytometric analysis of cell surface expression. Unstained cells and fluorescence minus one mAbs will be used as controls in all experiments. Lymphocytes will be identified first according to their light-scattering properties and then analysed for expression of lymphocyte markers. CD4+ and CD8+ T-cells will be defined as CD3+ CD4+ CD8- T-cells and CD3+ CD4- CD8+ T-cells, respectively. The associated expressions of CD45RA, CCR7 and CD27 will be then used to obtain the percentages of cells distinctively identifying naïve (CD45RA+ CCR7+ CD27+), central memory (CD45RA- CCR7+ CD27+), pre-terminal effector memory (CD45RA- CCR7- CD27-) and terminal effector memory (CD45RA+ CCR7- CD27-) CD4+ and CD8+ T-cell subsets. The expression of the CD38 activation marker will be then determined for each CD4+ or CD8+ T-cell subset. FlowJo data analysis software (FlowJo, LLC, Ashland, Oregon, United States) will be used for data analysis.

#### Th17 and Treg cell assessments

PBMCs will be cultured in 48-well culture plates at 0.5 to 1 × 10^6^ cells/mL per well and will be stimulated with 5 ng/mL phorbol 12-myristate 13-acetate (PMA) and 1 μg/ml ionomycin (both from Sigma Aldrich, Oakville, Ontario, Canada) for two hours at 37°C. A total of 2 μg/ml brefeldin A (Sigma Aldrich, Oakville, Ontario, Canada) will then be added to block cytokine secretion and cells will be cultured for 18 hours at 37°C. PBMCs will be surface stained followed by fixation and permeabilization using the Cytofix/Cytoperm Permeabilization kit (BD Bioscience, Mississauga, Ontario, Canada) for intracellular staining for IL-17A and IFN-ɣ (positive control). Treg cells will be defined by flow cytometry as being CD3+ CD4+ CD25^high^ CD127^low^ FoxP3^high^ and Th17 cells as being CD3+ CD4+ IL-17A+ upon PMA and ionomycin stimulation [27].

### Monocyte and dendritic cell immune activation

As IDO enzyme is expressed by monocytes and dendritic cells, we will also assess the impact of ER niacin on the activation of monocytes and dendritic cells (DCs). The expression of the activation markers CD69 and HLA-DR on CD14+ monocytes, as well as the expression of CD86 and CD83, markers of DC activation and maturation on both CD11c+ myeloid DCs and CD123+ plasmacytoid DC, will be evaluated by flow cytometry.

#### Inflammatory markers assessments

Although inflammatory markers such as D-dimer and hsCRP are related to cardiovascular non-AIDS events during HIV infection, it has become clear that HIV induces a hypercoagulable state as a consequence of impaired endothelial function despite ART. The effect of ER niacin on the levels of IFN-α, IL-1, IL-6, IL-17, IP-10, D-dimer and hsCRP, as well as on the microbial translocation markers sCD14 and LPS, will be assessed by Enzyme-Linked Immunosorbent Assay ELISA [51].

### Plasma Trp and Kyn assessments

Plasma levels of Trp and Kyn will be measured by an automated on-line solid-phase extraction-liquid chromatographic-tandem mass spectrometric (XLC-MS/MS) method as previously described [27, 53].

### Sample size and statistical analyses

Because this proof-of-concept study will explore a phenomenon with little *in vivo* data, a convenience sample of 20 participants was chosen without formal power calculations. The data that will be obtained should be sufficient to assist in such calculations for future large studies.

The primary comparison is the change in the percentage of CD3+ CD8+ HLA-DR+ T-cells between the period of ER niacin plus ART (from week 0 to week 24 of arm 1, and from week 24 to week 48 of arm 2) and the control periods of ART alone (from week 24 to 48 of arm 1, and from week 0 to 24 of arm 2). If the treatment effects in the two time periods look distinctly dissimilar, which may occur due to carryover effects of treatment, we will focus on the comparison between arms 1 and 2 at the end of 24 weeks.

In each time period, a between-groups t-test will be used to compare the change in the percentage of CD3+ CD8+ HLA-DR+ T-cells during the ER niacin plus ART treatment with the change in the percentage of CD3+ CD8+ HLA-DR+ T-cells in the ART treatment alone. If the results of these comparisons in the two treatment periods are similar, then within each treatment order, a paired t-test will be used to compare the change in the percentage of CD3+ CD8+ HLA-DR+ T-cells during the ER niacin plus ART period with the change in the percentage of CD8+ CD38+ T-cells during the ART-alone period and the results of these two comparisons will be combined to generate a pooled estimate of the treatment effect. The same analytic approach will be used for the secondary outcomes. The primary comparison would be based on 20 pairs of observations, with each subject receiving active ER niacin compared to the control time-points (ART alone without ER niacin) in different time-points. The ratio of the difference between the study groups to the standard deviation of the differences would have to be fairly large (0.625) to achieve a power of 80%. All statistical tests performed will be two-tailed with significance determined by reference to the 5% level, unless otherwise stated.

The same analytic approach will be used for the secondary outcomes as for the primary outcome. The secondary outcomes are: the change in total CD4+ T-cell count, changes in Th17 cells and Treg cells in blood and gut mucosa samples (from substudy), changes in cytokines and in inflammatory markers such as IFN-α, IL-1, IL-6, IL-17, IP-10, D-dimers, hsCRP, sCD14 and LPS, the change in Trp plasma levels, changes in monocyte and dendritic cell immune activation, changes in cholesterol and triglycerides and changes in QoL, fatigue, depression and neurocognitive scores, using computerized and pencil and paper tests (from optional procedures).

### Data management

All patient data and electronic files will be stored in the secure environment of the Chronic Viral Illness Service of the McGill University Health Centre, (Montreal, Quebec, Canada) and in the CTN database (Vancouver, British Columbia, Canada). All data will be anonymized for publication. Only researchers affiliated with the study will have access to participant data. Study progress and safety will be evaluated in an ongoing fashion by a CTN Data and Safety Monitoring Committee (DSMC).

### Storage of biological specimens

Biological samples will be stored at the Chronic Viral Illness Service of the McGill University Health Centre for analysis in the current trial and for future use in ancillary studies.

### Adverse events

The safety and tolerability of Niaspan FCT will be evaluated by vital signs and AE monitoring, both spontaneously reported by the participant and actively sought at each clinic visit. In addition, biological tolerability will be evaluated by hematology, biochemistry and any other clinical, laboratory or diagnostic tests conducted on the participants during the course of the study. The trial medical monitor and the trial investigator will receive and review the lab results for all participants for all assessed safety variables. The DSMC will also review safety information. The toxicity of Niaspan FCT will be assessed using the World Health Organization toxicity scale. Any AEs and laboratory abnormalities that occur during the study will be evaluated by the trial investigators and grade 3 and 4 AEs will be recorded on case report forms (CRFs). Grades 1 and 2 AEs specific to the effects of flushing will also be recorded on the CRFs. The ‘Bays-Ballantyne niacin-induced flushing and persistence of use questionnaire’ will be used during ER niacin administration to follow the participants’ perception of the side-effect of flushing [54]. If required, blood specimens will be collected for hematology and biochemistry tests. Participants having AEs will be monitored with relevant clinical assessments and laboratory tests, as determined by the trial investigator. The trial investigator will report ongoing AEs at completion of the clinical study to the primary care physician, who will determine the need for, and provide, standard medical care. The trial investigator will ensure that the event is satisfactorily resolved or that no further follow-up is required. Any participants who discontinue participation for an unresolved clinically significant AE will be followed until satisfactory clinical resolution is achieved and the AE recorded on the CRF, regardless of severity grade. AEs that may be related to ER niacin administration will be managed by dose reduction of the study drug and/or by taking 325 mg acetylsalicylic acid (ASA) 30 minutes prior to ER niacin administration and/or by dosing at bedtime [55]. In the case of life-threatening AEs, ER niacin will be discontinued permanently.

### Ethics

Written informed consent will be obtained from all study participants. The study protocol and consent forms have been approved by the Research Ethics Board of the McGill University Health Center (reference number: 11-019GEN) and by Health Canada’s Therapeutic Products Directorate (reference number: 9427-M2303-44C). The study will be conducted in accordance with the applicable Health Canada regulations, International Conference on Harmonization guidelines on current Good Clinical Practice and the Declaration of Helsinki.

## Discussion

ART therapy has significantly decreased HIV-associated morbidity and mortality over the last 15 years. Despite the substantial benefits of ART, immune reconstitution may not be complete for approximately 30% of patients who achieve virologic control [1, 2]. These patients remain at an elevated risk for clinical progression to both AIDS and non-AIDS events [1, 3–5].

We have designed a proof-of-concept study to assess supplementation with Niaspan FCT (an ER form of niacin) in combination with ART, compared to ART alone, on T-cell immune activation as defined by changes in the percentage of CD3+ CD8+ HLA-DR+ T-cells. A reduction in immune activation may improve CD4 recovery in HIV-infected individuals with suboptimal immune responses despite sustained virologic suppression. Our study also includes a neurocognition substudy that examines the effect of niacin on QoL, fatigue and depression.

It has been previously reported that a 10-fold difference exists between uninfected healthy controls and treated aviremic HIV-infected participants in the level of activated CD3+ CD8+ HLA-DR+ T-cells [52, 56]. It is expected that a 50% reduction in activated CD3+ CD8+ HLA-DR+ T-cells will be observed following ER niacin treatment which should translate into a clinically significant increase in CD4+ T-cell count (>100 CD4+ T-cells/μL). This estimate is based on a mean increase of 100 CD4+ T-cells/μL for participants having a two-fold reduction or not in their CD8+ T-cell activation level after 48 weeks of ART [57]. Assessments of activated CD4+ CD38+ and CD8+ CD38+ cell subsets will allow, for the first time, a more precise definition of which cell subpopulation(s) may change with ER niacin. Measurements of circulating cytokines, inflammatory mediators and LPS will be used to determine whether ER niacin administration can reduce or suppress inflammatory mediators known to drive immune activation.

Another objective of this study is to examine Niaspan FCT tolerance. Flushing at the initiation of niacin therapy is the most commonly reported side effect and is defined as redness, warmth, itching and/or a tingling sensation on the face, neck, chest and back [55]. This is a natural reaction signalling that niacin is in the bloodstream. Flushing is believed to be caused by an increase in blood flow and by the expansion of blood vessels close to the surface of the skin. Most participants on Niaspan FCT will experience this sensation, usually at the start of therapy or when the dosing is increased (occurring in up to 88% of participants in previous studies). These effects are usually transient and rarely require discontinuation of therapy (fewer than 6% discontinued due to flushing) [44]. Niaspan FCT, the drug to be administered in this pilot study, is an ER form of niacin that is dosed once each night when cholesterol synthesis and fatty acid mobilization are at their peak [38]. This form of niacin has intermediate-release characteristics and is thus designed to minimize both liver enzyme elevation and flushing. It is expected that for most participants, the flushing in response to ER niacin will occur over the first eight weeks of therapy and should become milder and less frequent as their bodies adjust to this drug.

For this pilot study, a convenience sample of 20 participants was chosen without formal power calculations. Should the results of this study suggest that niacin is tolerable, effective at reducing T-cell activation and/or has a positive effect on QoL, fatigue, depression and neurocognition, future studies will be performed. As a crossover study, two other comparisons will be made between the two arms: 1) the period of niacin plus ART in arm 1 will be compared to niacin plus ART in arm 2; 2) the period of ART alone in arm 1 will be compared to ART alone in arm 2. This will allow for the further evaluation of the potential continuity of the hypothesised effect of niacin following its discontinuation. The data obtained in this pilot study should be sufficient to assist in formal power calculations for such future studies.

### Dissemination plan

The results of the trial, regardless of outcome, will be disseminated through the traditional routes of scientific peer-reviewed publications, through international and national specialist conferences, and through the CTN. BL and MAJ will be responsible for initially drafting these manuscripts and professional writers will not be used for any of the publications. Authorship will be based on the criteria defined by the International Committee of Medical Journal Editors [58]. We aim to write the final results paper within six months of the un-blinding procedure. Subjects who have been involved in the trial will be given the option of having a summary of the results sent to them.

## Trial status

The trial was started in February 2012. We expect enrolment to be complete by November 2014.

## Electronic supplementary material

Additional file 1: **Participant Informed Consent.** (PDF 122 KB)

## Abbreviations

3-OHK: 3-hydroxy-kynurenine
AE(s): Adverse event(s)
AIDS: Acquired immune deficiency syndrome
ALT: Alanine transaminase
ART: Antiretroviral therapy
ASA: Acetylsalicylic acid
AST: Aspartate transaminase
CANTAB: Cambridge Neuropsychological Test Automated Battery
CES-D: Center for Epidemiologic Studies Depression scale
CRFs: Case report forms
DC(s): Dendritic cell(s)
DSMC: Data Safety Monitoring Committee
ER: Extended-release
FCT: Film coated tablets
HANDs: HIV-associated neurocognitive disorders
HIV: Human immunodeficiency virus
hsCRP: Highly sensitive C-reactive protein
HVLT-R: Hopkins Verbal Learning Test Revised
IDO: Indoleamine 2,3-dioxygenase
IFN-α: Interferon-alpha
IFN-γ: Interferon-gamma
IL: Interleukin
IP-10: Interferon gamma-induced protein 10
IR: Immediate-release
Kyn: kynurenine
LPS: Lipopolysaccharides
mAb(s): monoclonal antibody/antibodies
MUHC: McGill University Health Centre
PBMCs: Peripheral blood mononuclear cells
PMA: Phorbol 12-myristate 13-acetate
POMS: Profile of Mood State
QoL: Quality of life
RNA: Ribonucleic acid
SMAQ: Simple Medication Adherence Questionnaire
Th17 cells: T helper 17 cells
Treg cells: T regulatory cells
Trp: Tryptophan
TrpOx: Tryptophan oxidation
ULN: Upper limit of normal
WBC: White blood cell
WHOQOL-HIV BREF: World Health Organization quality of life instrument in patients with HIV infection brief
XLC-MS/MS: Extraction-liquid chromatographic-tandem mass spectrometry.
