# Supplementary material for: The role of extended-release niacin on immune activation and neurocognition in HIV-infected patients treated with antiretroviral therapy – CTN PT006: study protocol for a randomized controlled trial
Source: Trials. 2014 Oct 7;15:390. doi: 10.1186/1745-6215-15-390 (PMC4283109; doi:10.1186/1745-6215-15-390)
Supplement: Supplementary file 1 — Additional file 1: Participant Informed Consent. (PDF 122 KB) [file 13063_2014_2343_MOESM1_ESM.pdf]

PARTICIPANT INFORMED CONSENT

Study title: Role of extended-released niacin on immune activation in HIV-infected patients treated with antiretroviral therapy: a proof-of-concept study

Sponsor: MUHC and CIHR Canadian HIV Trials Network (CTN).

Principal investigator: Bertrand Lebouché, M.D. PhD, McGill University

Co-investigators:
Jean-Pierre Routy, M.D., McGill University
Richard Lalonde, M.D., McGill University
Norbert Gilmore, M.D., McGill University
Marina Klein, M.D., McGill University
Charles Frenette, M.D., McGill University
Alexandra de Pokomandy, M.D., McGill University
Roger LeBlanc, M.D., McGill University
Petronela Ancuta, PhD, Université de Montréal
Marie-Josée Brouillette, M.D., McGill University
Brian M. Gilfix, MDCM, PhD, McGill University
Simon N. Young, PhD, McGill University

Address of Study Site: Montreal Chest Institute
3650 St-Urbain Street, 8th Floor
Montreal (Quebec) H2X 2P4

INTRODUCTION

You are being asked to take part in the research study named above because you are infected with HIV, the virus that causes acquired immunodeficiency syndrome (AIDS). This is a study which will evaluate the effect of a drug called extended-released niacin (ER niacin) in individuals infected with HIV.

Before you decide to take part in this study, you should read the following document and ask any questions that you do not understand. Please take your time to make your decision about taking part. You may discuss your decision with your friends and family. You can also discuss it with your health care team. If you have any questions, you can ask your study doctor for more explanation.

BACKGROUND

Anti-HIV drugs can suppress viral replication and cut down on the number of serious infections that people with HIV get. In fact, there are a number of powerful anti-HIV drugs, which keep the virus at undetectable levels and enable HIV-infected individuals to live longer. However, some participants taking anti-HIV drugs do not achieve an adequate CD4 recovery (an increase in CD4 cell count) and remain at risk for developing AIDS and non-AIDS-related complications. Among factors associated with low CD4 recovery, T cell immune activation (CD4 and CD8 T cells expressing markers of activation such as CD38) represents one of the most important ones.

STUDY MEDICATION:

ER niacin (^{Pr}NiaspanFCT®) is an extended-released form of niacin, also known as vitamin B3. Niacin is effective in reducing cholesterol levels in the blood. This drug has been known for a long-time to treat dyslipidemia (high cholesterol and triglycerides in the blood) and it is used to improve favourably all the lipoprotein (assembly that contains protein and fat) risk factors for artherosclerotic disease (disease caused by build-up of fat in the arterial walls), particularly in HIV-infected patients. Recent scientific research shows that regular consumption of niacin-rich foods may also provide protection against Alzheimer's disease and age-related cognitive decline. ER niacin is taken by mouth.

ER niacin is an experimental drug and has *not been* approved by Health Canada for use in HIV-infected patients to reduce T cell immune activation and to improve neurocognitive functions.

PURPOSE OF THE STUDY

The purpose of this study is to find out:

1. If ER niacin combined with anti-HIV drugs, compared with anti-HIV drugs alone, could reduce T cell immune activation and enhance CD4 recovery;
2. If ER niacin can improve your quality of life and your neurocognitive functions

STUDY DESCRIPTION

Approximately 20 subjects will be enrolled in this study at the Montreal Chest Institute.

Your participation in this study will last about 48 weeks. In order to be eligible for the study, you must be 21 years of age or older, with undetectable viral load (the quantity of the HIV virus in the blood has to be less than 50 copies/ml) for at least 3 months and a CD4 count of less than 350 cells/µl.

Also, you will be asked to participate in an optional neurocognitive assessment. Please refer to the signature page of this informed consent document to confirm if you would like or not to participate in this sub-study.

You should tell your nurse or doctor before taking any natural health products (including supplements or herbal products) in addition to drugs, or enroll in other clinical trials. If

you are diabetic, inform your doctor if you notice any changes in your blood sugar. If you are taking another cholesterol lowering drug, you should inform your doctor if you experience any signs of muscle pain or weakness as this may be a sign of a rare but serious adverse drug reaction.

STUDY TREATMENT

Eligible participants will be assigned by chance (like the toss of a coin) to one of two treatment groups. You will have an equal (1 to 1) chance of being assigned to either one of the treatment group. The two groups are:

- Group 1: This group will receive an initial dose of ER niacin 500 mg by mouth, the first evening from week 0 to week 4 then increase it to 1000 mg once a day from week 5 to week 8, then increase to 1500 mg from week 9 to week 12 then increase to 2000 mg until weeks 24 and then stopped.

	**Weeks**	**Daily Dose**	**PrNiaspan FCT® Dosage**
Initial Titration Schedule	0 to 4	500 mg	one 500 mg tablet at bedtime
	5 to 8	1000 mg	one 1000 mg tablet or two 500 mg tablets at bedtime
	9 to 12	1500 mg	one 1000 mg tablet and one 500 mg tablet at bedtime
	13 to 24	2000 mg	two 1000 mg tablets at bedtime

- Group 2: This group will not receive ER niacin for the first 24 weeks. This group will receive an initial dose of ER niacin 500 mg the first evening at week 25 by mouth from week 25 to week 28 then increase it to 1000 mg once a day from week 29 to week 32, then increase to 1500 mg from week 33 to week 36 then increase to 2000 mg until week 48 and then stopped.

	**Weeks**	**Daily Dose**	**PrNiaspan FCT® Dosage**
Initial Titration Schedule	25 to 28	500 mg	one 500 mg tablet at bedtime
	29 to 32	1000 mg	one 1000 mg tablet or two 500 mg tablets at bedtime
	33 to 36	1500 mg	one 1000 mg tablet and one 500 mg tablet at bedtime
	37 to 48	2000 mg	two 1000 mg tablets at bedtime

Both groups must continue to take their anti-HIV drugs during the 48 week study period (i.e.. from Week 0 to Week 48).

STUDY PROCEDURES

Eight weeks prior to being enrolled into the study, you will be assessed to determine if the HIV virus in your blood meets the eligibility criteria to take part in this study. If you do not meet all the eligible criteria, you will be excluded from the study.

If you are eligible and agree to take part in this study, you will be asked to return to the clinic at the start of the study (week 0) and subsequently at weeks 4, 8, 12, 16, 20, 24 and 48 if you are in Group 1, or at weeks 24, 28, 32, 36, 40, 44, and 48 in you are in Group 2. At these visits you will receive ER niacin (progressively to 2000 mg once a day). ER niacin will be taken by mouth at bed time. You must continue to take your anti-HIV drugs during the 48-week study period (i.e. form Week 0 to Week 48).

Screening Visit (Week -8 to -1)

The first study visit will last approximately 30 minutes you will undergo the following procedures:

- You will be asked about your demographics information, your medical history including concomitant medications (all medication that you are currently using) and ART medication you are taking
- A complete physical examination including vital signs assessment and measure of weight and height
- A urine pregnancy test (for women able to become pregnant) will be done
- About 70 ml (15 teaspoons) of blood will be taken for the following tests:
    - Measure CD4 and CD8 cell counts,
    - Measure plasma HIV RNA levels
    - Perform haematology and fasted biochemistry tests
    - Testing for Hepatitis B and C virus
    - Testing for Syphilis virus
-

The Investigator will inform you that the information gathered at this Screening Visit will be reviewed and you will be contacted to attend a Baseline Visit if eligibility requirements are met. If you are eligible, you will be given the following instructions to prepare for each visit:

- fast for 8 hours prior to the visit
- refrain from consuming non-prescription drugs (except for over-the-counter medications) 48 hours prior to the Baseline, Week 24 and Week 48 visits
- refrain from marijuana use 24 hours prior to the Baseline, Week 24 and Week 48 visits
- refrain from consuming any alcohol 24 hours prior to the Baseline, Week 24 and Week 48 visits, other than 1 drink at the previous night's meal.

Baseline visit (Week 0)

During this visit, you will undergo the following procedures:

- You will be asked about your medical history including concomitant medications (all medication that you are currently using) and ART medication you are taking
- A complete physical examination including vital signs assessment and measure of weight
- About 75 ml (15 teaspoons) of blood will be taken for the following tests:
    - Measure CD4 and CD8 cell counts
    - Measure plasma HIV RNA levels
    - Perform fasted biochemistry tests
    - Assess complete blood count
    - Assess T cell activation and inflammatory markers.
    - Testing levels of tryptophan, niacin and its derivatives
- Complete a diet survey (with a dietician) to determine the pre-study dietary niacin levels
- Adverse events will be assessed
- You will be asked if you would like or would not like to complete 5 optional questionnaires about your health status and your quality of life (neurocognitive assessment), which will take approximately 2 hours and 30 minutes: WHOQOL-BREF (for quality of life), CES-D (for depression), POMS (for mood state), HVLT-R (for verbal leaning evaluation) and CANTAB® (for neurocognitive evaluation). As stated previously, see the signature page of the informed consent document regarding completion of these questionnaires.
- Complete the Simplified Medication Adherence Questionnaire (SMAQ) to evaluate your adherence to ART. This questionnaire will take approximately 10 minutes to complete.
- ER niacin dispensation will be performed (only for Group 1)

Follow-up visits (Week 4, Week 8, Week 12, Week 16, Week 20, Week 24 and Week 48 for Group 1 and Week 24, Week 28, Week 32, Week 36, Week 40, Week 44 and Week 48 for Group 2)

During these visits, you will undergo the following procedures:

- Perform a physical exam targeted to the dermatological effects of flushing including vital signs assessment and measure of weight.
- A urine pregnancy test (for women able to become pregnant) will be done (only at Week 24 for Group 2)
- About 75 ml (15 teaspoons) of blood will be taken for the following tests:
    - Hematology and biochemistry tests
    - Measure CD4 and CD8 cell counts
    - Measuring plasma HIV RNA levels
    - Testing levels of tryptophan, niacin and its derivatives
    - Testing T-cell activation and inflammatory markers
    - Testing Syphilis at Weeks 24 and 48 if participant tested positive at Baseline
- You will be asked to complete the Bays-Ballantyne questionnaire. This questionnaire will be used during ER niacin administration to follow your perception of the side effect of flushing and will take 5 minutes to complete

- If you agreed to participate in the neurocognitive assessment, you will be asked to complete the 5 optional questionnaires about your health status and your quality of life, which will take approximately 2 hours and 30 minutes (only at Weeks 24 and 48): WHOQOL-BREF (for quality of life), CES-D (for depression), POMS (for mood state), HVLT-R (for verbal leaning evaluation) and CANTAB® (for neurocognitive evaluation)
- Complete the Simplified Medication Adherence Questionnaire (SMAQ) to evaluate your adherence to treatments (only at 3 study visits)
- Adverse events will be assessed
- Evaluation of concomitant medications (all medication that you are currently using).
-
- ER niacin dispensation will be performed.

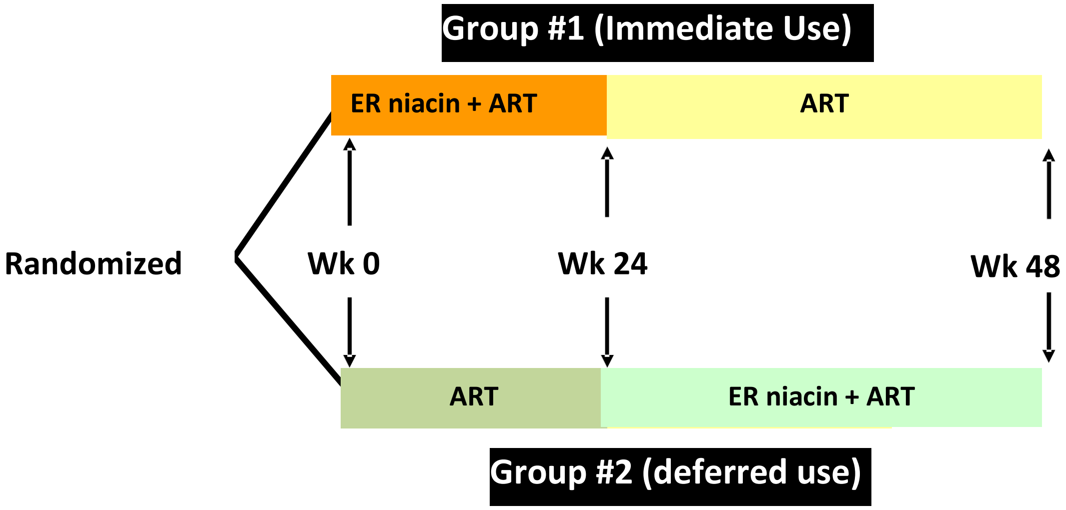

If you miss your study visits, with your permission, a person from the clinic will try to contact you. Even participants who stop taking ER niacin for any reason will be asked to complete the follow-up visits. Also, if you decide to stop taking part in this study, you will be asked if the research nurse may contact you for follow-up visits until week 48. Clinic personnel will ask you questions to see how you have been doing since stopping the study, as a safety measure. Clinic personnel trying to contact you will be careful to maintain your privacy and will not tell anyone, without your permission, about you taking part in an HIV study. If you develop side effects from ER niacin, your doctor may want to see you more often. Additional blood samples may be needed.

POTENTIAL RISKS AND/OR DISCOMFORTS

The most commonly reported side effects at the initiation of ER Niacin is redness, warmth, itching, and/or a tingling sensation on the face, neck, chest and back, that doctors refer to a flushing. This is a natural reaction signaling that ER niacin is in the

bloodstream. Flushing is believed to be caused by an increase in blood flow and the expansion of blood vessels close to the surface of the skin. Most patients on ER niacin will experience this sensation, usually at the start of therapy or when the dosing is increased (occurring up to 88% of patients). These effects are usually transient and rarely require discontinuation of therapy (fewer than 6 % discontinued due to flushing). For most patients, the flushing occurs over the first 8 weeks of therapy and will become milder and less frequent as your body adjusts to ER niacin. If flushing occurs, it usually does so within 2 to 4 hours after taking NIASPAN and may last for a few hours.

Flushing of the skin may be reduced in frequency or severity with a small snack (e.g., 2 crackers) or by 325 mg dose of aspirin 30 minutes prior to ER niacin dosing.

By dosing at bedtime, flushing will most likely occur during sleep. However, if awakened by flushing at night, you should get up slowly, especially if feeling dizzy, feeling faint, or taking blood pressure medications.In some patients, flushing may be more intense. Additional symptoms, such as rapid or pronounced heartbeat or dizziness, shortness of breath, sweating, chills, and/or swelling may occur; on rare occasions, fainting may occur. If the flushing wakes you up and you wish to get out of bed, take your time and get up slowly - especially if you start to feel faint or dizzy, or if you take blood pressure medication.

All participants are invited to reach the dose of 2000 mg daily of ER niacin. Those who will be unable to tolerate a dosage increase will continue at the previously tolerated dose for the duration of the study.

Other than flushing, the side effects most often seen are stuffy nose (31%), gastrointestinal in nature, such as stomach upset (13%), nausea (13%) and diarrhea (20%); pain back (9%), rash and itching also are occasionally observed.

A recently completed study enrolled over 25 000 patients with pre-existing cardiovascular disease and considered at high risk for cardiovascular events. All patients received a statin (to lower the ‘bad’ cholesterol) and were randomly assigned to receive treatment with an ER niacin-laropiprant combination or placebo for about 4 years. Of those assigned to the ER niacin-laropiprant combination treatment reported unexpected side effects included infections and bleeding (mainly in the gut and brain). It was also noted that ER niacin/laropiprant also increased the risk of occurrence of the rare side effect of statins called “myopathy” which is caused by muscle breakdown. Myopathy causes weak and painful muscles and rarely is severe enough to damage the kidneys.

Precaution

Severe liver damage can occur when switching to long-acting niacin (NIASPAN) from immediate-release niacin. All forms of niacin are not the same as NIASPAN. You will not be able to switch between forms of niacin without talking to your health care provider.

Pregnancy and childbearing potential

No information is available on the safety of ER niacin in pregnant women. If you become pregnant during your participation in this clinical study, the drug should be discontinued.

Therefore, you cannot take part in this study if you are breastfeeding, pregnant, think that you may be pregnant, or trying to get pregnant. All fecund female participants (women of child-bearing potential) will be tested for pregnancy during the trial. The term fecund female participants refers to women of child-bearing potential, which means women that can become pregnant and excludes women population defined as either post-menopausal (12 months of spontaneous amenorrhea and 45 years of age) or physically incapable of becoming pregnant with documented tubal ligation, hysterectomy or bilateral oophorectomy.

If you become pregnant during your participation in this clinical study, the drug should be discontinued; you will be discontinued from the study, and referred for obstetrical care. If you become pregnant or think you are pregnant, tell the study doctor immediately. All study participants must agree to use one of the following methods of contraception to avoid pregnancy:

- Complete abstinence from intercourse from 2 weeks prior to administration of IP, throughout the study, and for at least 2 weeks after discontinuation of all study
- Double barrier method (male condom/Intrauterine device, male condom/diaphragm, diaphragm/spermicide, male condom/cervical cap).
- Any intrauterine device (IUD) with published data showing that the expected failure rate is <1% per year
- Any other method with published data showing that the expected failure rate is <1% per year.

When using a condom, it is recommended to use a condom without spermicide.
The cervical cap is an inferior contraceptive barrier, especially in women who have given birth at least once. Therefore, if you are a woman who has given birth, please speak with the study doctor about birth control methods that you can use.

If you are a female who is sexually active and able to become pregnant, please speak with the study doctor about what kind of birth control methods to use and how long to use them.

Men
Because the effect of study medication on sperm is unknown, pregnancies in partners of male subjects need to be reported within 24 hours.

If your spouse or partner thinks she is pregnant during the study or within 28 days after you have stopped taking niacin, tell your study doctor immediately.

Drug-Food Interactions
Spicy foods and hot or alcoholic beverages, if took together may increase the side effects of flushing and pruritus and should be avoided around the time of taking ER niacin.

Drug interactions
ER niacin has a low potential for drug-drug interactions as it is not known to stop or increase the metabolism of other medications. Niacin absorption may be reduced if took at the same time as cholestyramine or colestipol. If these drugs are part of your medication, you must take those 4 to 6 hours before or after niacin.

An increased risk of myopathy and rhabdomyolysis (breakdown of muscle fibers resulting in the release of muscle fiber contents (myoglobin) into the bloodstream) has been reported by patients who were taking niacin with HMG-CoA reductase inhibitor (e.g. statin) or fibrate classes (for the control of cholesterol). If you are receiving these medications, you can be part of this study, but you will be closely followed for these adverse reactions.

Niacin can also increase the risk of glucose intolerance associated with protease inhibitors. In addition, niacin can decrease serum phosphate and increase HDL cholesterol in dialysed participants. Therefore, creatine kinase (CK), fasting glucose, HDL, and serum phosphate will be monitored closely.

Niacin may potentiate the effects of ganglionic blocking agents and vasoactive drugs resulting in postural hypotension.

Vitamins or other nutritional supplements containing large doses of niacin or related compounds such as nicotinamide may potentiate the adverse effects of ^{Pr}Niaspan FCT®.

Risks of blood testing
The risks of having blood taken are less than 1% and include discomfort, bleeding, or bruising where the needle enters the body.

NEW INFORMATION

If any new information becomes available during the study that could affect your willingness to continue to participate, it will be supplied to you.

POTENTIAL BENEFITS

We do not know whether being in this study will benefit you. However, even if it does, we do not know whether a CD4 recovery will improve your health. What we learn from this study may help us to improve your own treatment and the treatment of other people who are infected with HIV. At the end of the study, you will be told when study results may be available and how to learn about them.

ALTERNATIVE TO STUDY RESEARCH

You do not have to join this study to receive treatment for your condition. In addition, there may be other research studies looking at other ways of managing this stage of HIV infection. Your doctor will discuss any of these options with you. If the guidelines for HIV therapy change during the course of this study, you will be advised

COSTS
There will be no cost to you for participating in this study. All clinical and professional services, diagnosis and laboratory works that are part of this research will be provided to you at no cost. The ER niacin (^{Pr}NiaspanFCT®) will be supplied to you at no cost. You will continue to obtain your other anti-HIV treatment by prescription and the Government drug program or your own insurance will pay it for.

REIMBURSMENT
You will be compensated for your travel and childcare up to a maximum of $25.00 per visit.

CONFIDENTIALITY
If you decide to be in this study, the study doctor and research team will collect information that identifies you (or personal information). This may include your name, address, phone number, health plan number, date of birth, medical history, and medical-related information, which shall be collected from your family doctor (or other health care workers) during your study visits. All personal information obtained during this study will be kept confidential. All information collected from you will be kept at the study site and reasonable safeguards will be put into place to protect your information.

The research team will consult your medical file to collect information relating to your medical history and take notes of the relevant data for this research project. All study data will be sent to the Sponsor and / or its representatives, as long as they agree to use the information as described here, however this data would not identify you by name. Your name will be coded and the code list will be kept in a locked filing cabinet at the study site with limited access.

Your study data might be provided to the following groups, if required:

- Representatives of the Sponsor
- Government regulatory agencies, including Health Canada, the United States FDA and similar government agencies from other countries;
- MUHC-MGH Research Ethics Board (people who review the research study to protect your rights);
- Any other entity as required by law.

To verify the research data, other physicians participating in this research study at this institution and monitors from the following organizations may review your original medical chart (contains information that can directly identify you) for quality assurance and data analysis:

- The Sponsor or its representatives (e.g. clinical monitors and auditors) may inspect research and medical records in the presence of the investigator or study staff, however, they will not be able to record any information and no such records will be allowed to leave the investigator's office;
- The Quality Assurance Officer from the MUHC-MGH Research Ethics Board;
- Government regulatory agencies, including Health Canada, the United States

FDA, or their authorized representatives. Information may be provided to these agencies in a way that maintains your privacy according to United States and Canadian regulations.

By signing this consent form, you give us permission to release and disclose your personal health information as described above, and to inform your primary treating physician of your study participation. The results from this study may be published, however your identity will not be revealed in the combined results. Your confidentiality will be protected to the extent permitted by applicable laws and regulations.

CONTROL OF ETHICAL ASPECTS OF THE RESEARCH PROJECT
The Research Ethics Board of the MUHC approved this research project and ensures the follow-up. In addition, it will first approve any review and amendment made to the information/consent form and to the study protocol.

INDEMNIFICATION/ COMPENSATION IN CASE OF INJURY
If you should suffer any injury following your participation in the research project, you will receive the appropriate care and services for your medical condition without any charge to you.No other form of compensation will be awarded for injuries or complications related to this research. However, by signing this consent form, you are not waiving any of your legal rights nor are you freeing the investigators, sponsors, or the health establishment where the study takes place from their legal and professional responsibilities (this will be stated on the signature page.)

The McGill University Health Centre, the MUHC Research Institute, and investigators would not be able to offer compensation in the unlikely event of any injury resulting from your participation in this research study. However, by accepting to participate in this project, you are not waiving any of your legal rights nor discharging the researchers, the granting agency or the institution of their civil and professional responsibility.

VOLUNTARY PARTICIPATION AND STUDY WITHDRAWAL
Your participation in this study is voluntary. You are free to withdraw from the study at any time. Please inform the study staff if you decide to withdraw because you will be asked to come to the clinic for a termination visit and your health should be monitored. If you decide not to participate in the study or withdraw from it, your health care will not be affected. You may refuse to answer any question you do not want to answer, or not answer an interview question by saying “pass”. During the course of the study, you will be kept informed of any new treatments or findings that may influence your participation in the study. It is your responsibility to follow the directions and rules of the study. In addition, your participation in the study could be terminated by your doctor because of side effects from the drug, failure to take the drug as indicated or to come for study visits or if the study is stopped for other reasons. If this occurs, you will be given full explanation.

BLOOD AND MUCOSA SAMPLE BANKING
Your blood samples contain several types of cells that will not be possible to analyze immediately after the collections for several reasons. The tests are expensive and need to

be batched. Your collected samples will be stored under the supervision of Drs. Lebouché and Routy at the Montreal Chest Institute. The storage period for your samples will be for 15 years, after that your samples will be destroyed. During this period you can ask Drs. Lebouché or Routy to withdraw your stored samples from the bank. Only the researcher who will perform the analysis will have access to your samples. Blood samples will be used only for the purposes of this HIV-research study and no additional tests (e.g. genetic tests) will be performed on these samples without additional consent. Research results will not be returned, either to participants or to their physicians.

QUALITY ASSURANCE PROGRAM

The MUHC implemented a Quality Assurance Program that includes active continuing review of projects (on site visits) conducted within our establishment. Therefore, it must be noted that all human subject research conducted at the MUHC or elsewhere by its staff, is subject to MUHC Routine and Directed Quality Improvement Visits.

FUNDING OF THE RESEARCH PROJECT

This research study is being funded by the Canadian HIV Trials Network (CTN), and will be run by Dr. Bertrand Lebouché. The study doctor is being awarded money for including you and looking after you during your participation in this study.

INFORMATION AND CONTACTS

- If you have questions about this clinical research study, you may contact**, Dr. Bertrand Lebouché** during working hours at **(514) 934-1934 extension 32600**.

- If you have questions concerning your rights as a Research Participant and wish to discuss them with someone not connected to the clinical research study, please contact the **Ombudsman of the McGill University Health Centre (514) 934-1934 extension 35655**.

- If you believe you have been injured as a result of participating in this study, you may contact the **Director of Professional Services at (514) 934-1934, ext. 34329**

Study Title: Role of extended-released niacin on immune activation in HIV-infected patients treated with ART: a proof-of-concept study

PARTICIPANT INFORMED CONSENT
SIGNATURE PAGE

I have read, or have had read to me, this 13-page informed consent form and I voluntarily agree to participate in this research study, understanding that I may withdraw my participation at any time. I have had the opportunity to ask questions and all of my questions have been answered to my satisfaction. I have been given sufficient time to consider the above information and to seek advice. I will be given a copy of this signed and dated Informed Consent Form. By signing this consent form, I am not giving up any of my legal rights.

☐ I **agree** to participate in the sub-study on neurocognitive assessment.

☐ I **don't agree** to participate in the sub-study on neurocognitive assessment.

______________________	________________________	__________
Signature of Participant	Name (in block letters)	Date
______________________	________________________	__________
Signature of designate administering Informed Consent	Name (in block letters)	Date
______________________	________________________	__________
Signature of Investigator/ Co-investigator	Name (in block letters)	Date
